# University of Niagara

**Published:** 1898-06

**Authors:** 


A Monthly Review of Medicine and Surgery.

*EDITORS:*

THOMAS LOTHROP, M. D. - - WM. WARREN POTTER, M. D.

All communications, whether of a literary or business nature, should be addressed to the managing editor: 284 FRANKLIN STREET, BUFFALO, N. Y.

VOL. XXXVII. JUNE, 1898. NO. 11

### UNIVERSITY OF NIAGARA.

### ANNUAL COMMENCEMENT OF THE MEDICAL DEPARTMENT.

THE University of Niagara observed the thirteenth annual convocation of its medical department on Wednesday, May 11, 1898, at which ten candidates for the degree of doctor of medicine received their diplomas. The board of medical examiners assembled in the morning and tested the training the candidates had received and the value of their equipment to practise the healing art. The following-named members of the board were present: Dr. Frank W. Ross, of Elmira; Dr. Nelson G. Richmond, of Fredonia; Dr. Bentley S. Bourne, of Hamburg; Dr. Julius H. Potter and Dr. L. J. McAdam, of Buffalo. This board conducts its examination independently of and apart from the faculty, its action is final and no candidate whom it rejects can receive his degree. It is stated upon good authority that the graduating class of 1898 passed an uncommonly fine examination.

### ALUMNI ASSOCIATION.

The annual meeting of the alumni association was held in the college building on Wednesday, May 11, beginning at 10.30 A. M., at which time Dr. John A. Miller, professor of chemistry, delivered an address of welcome on behalf of the faculty. The routine business of the association was then disposed of. The president of the association, Dr. Frank W. Maloney, of Rochester, delivered his address at the opening of the afternoon session, when he discussed the subject of Rheumatism. Dr. M. Hartwig presented a paper entitled Some remarks about incipient orthopedic cases. Dr. William G. Ring, of Buffalo, read a paper entitled Movable kidney. Dr. L. Pierce Clark, of the Craig epileptic colony, Sonyea, N. Y., discussed the Pathogeny of an epileptic fit. Dr. L. J. McAdam, of Buffalo, also presented a paper, the subject of which was Remarks on appendicitis with report of cases.

The officers elected for the ensuing year are as follows: President, Dr. William G. Taylor, of Buffalo; vice-presidents, Drs. Frank W. McGuire and Charles E. Congdon, of Buffalo; secretary, Dr. J. Glen Ernest, of Buffalo; executive committee, Drs. J. J. Finerty, E. A. Forsyth and E. E. Koehler, of Buffalo. The alumni meetings were well attended and a growing interest was manifested in its scientific work as well as in the prosperity of the college. This interest was stimulated in a great measure by the executive committee, whose labors, though arduous, were performed with undiminished enthusiasm. The committee of 1898 consisted of Drs. J. J. Finerty, J. Henry Dowd and H. C. Buswell, who are entitled to great credit for the success of the meeting and for the most excellent banquet served at the Genesee hotel in the evening.

### COMMENCEMENT EXERCISES.

The graduating exercises were held at Concert Hall in the evening of commencement day beginning at 8 o'clock. A vast assemblage filled the spacious hall at an early hour, eager to witness the interesting ceremonies and to do honor to the young men who were about to receive their degrees in medicine, as a testimony of four years of hard and earnest work in the acquirement of an honorable profession.

The ceremonies were unusually impressive, for in the conferring of the degrees the university custom of hooding the candidates was observed. When the curtain was raised the faculty of the college was disclosed, already seated. The members of the class, attired in caps and gowns, then took the seats reserved for them in the front and center of the auditorium.

Dr. Eugene A. Smith had been appointed to deliver an address on the part of the faculty, but his duties as an officer of the 65th regiment, N. G. N. Y., made his attendance necessary at the camp of the regiment at Hempstead, preparatory to marching to the seat of war. In his stead the Rev. P. McHale, president and vice-chancellor of the university, delivered the opening address.

The speaker explained that a degree was an ending of one period in learning and the beginning of another. He counseled the graduates to remember that the fundamental idea of the calling of medicine was love of humanity regardless of race, nation, religion or profession.

He paid tribute to the memory of the late Dr. John Cronyn, founder and former president of the medical faculty.

During his address the vice-chancellor read a letter from Miss Elizabeth Cronyn, written on behalf of her mother, congratulating the faculty on the fifteenth anniversary of the founding of the university. With the letter were sent fifteen beautiful roses, one for each year of the life of the institution.

Following his address Vice-Chancellor McHale conferred the degree of Doctor of Medicine upon the following-named graduates: Albert Edward Brennan, of Cheektowaga; Charles Aaron Brownell, Bruce Leon De Witt Cook, Michael Aloysius Sullivan, Clarke Wallace Stewart and William Thomas Griffin, of Buffalo; George Henry Grant, of Rochester; Albert Joseph Lawler, of Elmira; James Munsie of Toronto, Ont., and Richard Edward Sweeney, of New York.

The oath of Hippocrates was administered to the class and Dr. H. C. Buswell then presented each candidate to the vice-chancellor, repeating in each instance a Latin formula to the effect that the applicant was entitled to the honor of the degree. The candidate knelt before the vice-chancellor, who conferred the degree of a full graduate of the university, Dr. John D. Flagg performing the duty of hooding the graduates. The candidates one after another arose, signed the roll and received their diplomas at the hands of Dr. A. A. Hubbell.

The Rev. Charles C. Albertson made the address of the evening, in the course of which he referred to Admiral Dewey and his fight against the Spaniards as a contest between modern and decadent ideas, a sentiment which the audience applauded with enthusiasm. The orator pleaded with the class to preserve clean personal characters and to go out into the world with a deep feeling of the responsibility and honor of the profession.

### THE BANQUET.

At the conclusion of the commencement exercises the alumni, faculty, new graduates and guests repaired to the Genesee hotel, where the annual dinner soon afterward was served. Among those present from out of town were Dr. F. W. Ross, of Elmira; Dr. Nelson G. Richmond, of Fredonia; Dr. Frank W. Maloney, of Rochester, and Dr. M. B. Shaw, of Eden.

Covers were laid for about 70 persons and seats at the tables were taken at 10 o'clock. While coffee was serving, the president, Dr. Frank W. Maloney, of Rochester, who acted as toastmaster, called the assemblage to order and after a few introductory remarks announced the speakers and subjects as follows :

Niagara University, Rev. L. A. Grace; What a Doctor knows about running a city, Dr. Conrad Diehl; War, Rev. C. C. Albertson; My Experience with Malpractice Cases, Hon. Joseph B. Brien; Newspaper Medicine, John J. O'Brien; Alumni, P. H. Hourigan, M. D.

The absence of Mayor Diehl, who was visiting the 65th regiment at Hempstead, and of the Rev. C. C. Albertson, who was engaged elsewhere, rendered it necessary to somewhat modify the printed program, but Dr. F. W. Ross, of Elmira, and others were summoned to fill the breach and when adjournment finally came, some time after midnight, partings were said amidst praises for the officers and committees who had contributed their services so generously and efficiently toward the consummation of a delightful day and evening.

